# 435. Clinical, Radiographic, and Physiological Correlates of post-COVID-19 Respiratory Symptoms: Results from the Chronic Impairment with Pulmonary Symptoms (ChIPS) Sub-study

**DOI:** 10.1093/ofid/ofad500.505

**Published:** 2023-11-27

**Authors:** S Michael Goertzen, David Lindholm, Robert Walter, Nikhil Huprikar, Anuradha Ganesan, Stephanie A Richard, Katrin Mende, Christine Murillo, Travis Harrell, P Gabriel Peterson, Mark P Simons, Robert O’Connell, David Tribble, Brian Agan, Timothy Burgess, Simon Pollett, Michael Morris

**Affiliations:** Brooke Army Medical Center, San Antonio, Texas; Department of Medicine, Uniformed Services University of the Health Sciences; Brooke Army Medical Center, San Antonio, Texas; Brooke Army Medical Center, San Antonio, Texas; Walter Reed National Military Medical Center, Bethesda, Maryland; Infectious Disease Clinical Research Program, USUHS; Henry M. Jackson Foundation for the Advancement of Military Medicine Inc, Bethesda, Maryland; Infectious Disease Clinical Research Program, Department of Preventive Medicine and Biostatistics, Uniformed Services University of the Health Sciences, Bethesda, MD, USA, Bethesda, Maryland; 1Infectious Disease Clinical Research Program, Department of Preventive Medicine and Biostatistics, Uniformed Services University of the Health Sciences and Brooke Army Medical Center, JBSA Fort Sam Houston, TX, San Antonio, TX; Infectious Diseases Clinical Research Program, USUHS; Henry M. Jackson Foundation for the Advancement of Military Medicine Inc, San Antonio, Texas; Walter Reed National Military Medical Center, Bethesda, Maryland; Walter Reed National Military Medical Center, Bethesda, Maryland; Infectious Disease Clinical Research Program, Department of Preventive Medicine and Biostatistics, Uniformed Services University of the Health Sciences, Bethesda, MD, USA, Bethesda, Maryland; Infectious Disease Clinical Research Program, USUHS, Bethesda, Maryland; Infectious Disease Clinical Research Program, Department of Preventive Medicine and Biostatistics, Uniformed Services University of the Health Sciences, Bethesda, MD, USA, Bethesda, Maryland; Infectious Disease Clinical Research Program, Department of Preventive Medicine and Biostatistics, Uniformed Services University of the Health Sciences, Bethesda, MD, USA, Bethesda, Maryland; Infectious Disease Clinical Research Program, Department of Preventive Medicine and Biostatistics, Uniformed Services University of the Health Sciences, Bethesda, MD, USA, Bethesda, Maryland; Infectious Disease Clinical Research Program, Department of Preventive Medicine and Biostatistics, Uniformed Services University of the Health Sciences, Bethesda, MD, USA, Bethesda, Maryland; Brooke Army Medical Center, San Antonio, Texas

## Abstract

**Background:**

The pathology of persistent post-COVID-19 symptoms remains poorly understood. We examined radiographic and physiological correlates in those with ongoing post-COVID-19 dyspnea compared to those with resolved dyspnea.

**Methods:**

The Epidemiology, Immunology, and Clinical Characteristics of Emerging Infectious Diseases with Pandemic Potential (EPICC) is a COVID-19 cohort study of Military Health System (MHS) beneficiaries. Study participants aged 18-65, with no pre-existing significant cardiopulmonary disease, and respiratory symptoms ≥ 3 months after COVID-19 onset were enrolled into the ChIPS sub-study as cases. Controls with resolved post-COVID-19 symptoms were also enrolled. Each participant underwent high resolution chest CT (HRCT), transthoracic echocardiography (TTE), electrocardiogram (ECG), full pulmonary function testing (PFT), impulse oscillometry (IOS), and a six minute walk test (6MWT) with Borg dyspnea scale.

**Results:**

There were 115 participants enrolled in the ChIPS sub-study, of whom 39 had persistent dyspnea (cases) and 76 had resolved dyspnea (controls) (Table 1). There was no statistically significant difference in age, sex, comorbidity index, or infecting variant between cases and controls. Cases were more likely to be unvaccinated at the time of initial infection. The average FEV_1_/FVC, FVC and FEV_1_ was within normal ranges for both cases and controls, though mean FEV_1_/FVC was higher in those with persistent dyspnea (Table 2). DLCO and IOS were similar between groups (Table 2). Cases had a decreased 6MWT distance and higher post-6MWT Borg scores compared to controls (Table 2). There were no significant differences in TTE results between groups. Variable ECG changes were seen in both groups with no statistically significant differences. HRCT findings are currently being analyzed.

**Figure:**
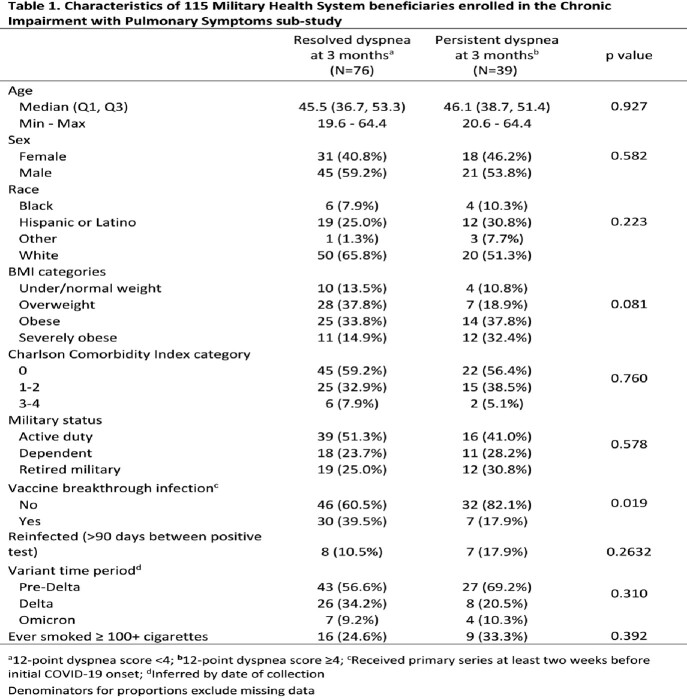

**Figure:**
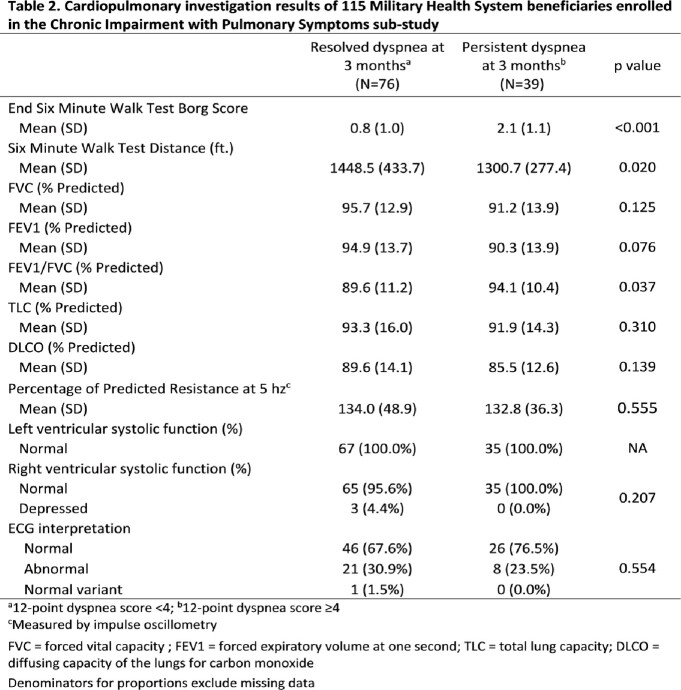

**Conclusion:**

We noted a small decrease in 6MWT distance and increased post-exertional Borg scores in those with persistent post-COVID symptoms; these should be explored as interventional study endpoints. PFT, IOS, TTE, and ECG findings were similar between groups. Those with vaccine-breakthrough infections were less likely to have persistent dyspnea.

**Disclosures:**

**Mark P. Simons, PhD**, AstraZeneca: The IDCRP and HJF were funded to conduct an unrelated phase III COVID-19 monoclonal antibody immunoprophylaxis trial as part of US Govt COVID Response **David Tribble, MD, DrPH**, AstraZeneca: The IDCRP and HJF were funded to conduct an unrelated phase III COVID-19 monoclonal antibody immunoprophylaxis trial as part of US Govt COVID response **Timothy Burgess, MD, MPH**, AstraZeneca: The IDCRP and the Henry M. Jackson Foundation (HJF) were funded to conduct an unrelated phase III COVID-19 monoclonal antibody immunoprophylaxis trial **Simon Pollett, MBBS**, AstraZeneca: The IDCRP and the Henry M. Jackson Foundation (HJF) were funded to conduct an unrelated phase III COVID-19 monoclonal antibody immunoprophylaxis trial **Michael Morris, MD**, Janssen Pharmaceuticals: Paid speaker (unrelated to this project and COVID-19 in general)

